# Supplementary material for: Representational learning by optimization of neural manifolds in an olfactory memory network
Source: Nat Neurosci. 2026 Sep 10;29(11):2810–21. doi: 10.1038/s41593-026-02429-3 (PMC13724268; doi:10.1038/s41593-026-02429-3)
Supplement: Supplementary file 1 [file 41593_2026_2429_MOESM1_ESM.pdf]

nature neuroscience

Supplementary information

https://doi.org/10.1038/s41593-026-02429-3

Representational learning by optimization of neural manifolds in an olfactory memory network

In the format provided by the authors and unedited

Supplementary Information

Representational learning by optimization of neural manifolds in an olfactory memory network

Bo Hu[1,2]*, Nesibe Z. Temiz[1,2]*, Chi-Ning Chou[3], Peter Rupprecht[1,4,5], Claire Meissner-Bernard[1], Benjamin Titze[1], SueYeon Chung[3,6], Rainer W. Friedrich[1,2]

1 Friedrich Miescher Institute for Biomedical Research, Fabrikstrasse 24, 4056 Basel, Switzerland
2 University of Basel, 4003 Basel, Switzerland
3 Center for Computational Neuroscience, Flatiron Institute, New York, NY, USA
4 Neuroscience Center Zurich, 8057 Zurich, Switzerland
5 Brain Research Institute, University of Zurich, 8057 Zurich, Switzerland
6 Center for Neural Science, New York University, New York, NY, USA
* These authors contributed equally to this work

Corresponding author: Rainer W. Friedrich, Friedrich Miescher Institute for Biomedical Research, Fabrikstrasse 24, 4056 Basel, Switzerland; rainer.friedrich@fmi.ch

- **Supplementary Note 1**: Manifold Capacity and Effective Geometric Measures
- **Supplementary Note 2**: Detailed information on statistical analyses shown in figures
- **Supplementary Note 3**: Manifold capacity analysis in the OB
- **Supplementary Table 1**: Weight strength parameter settings

Supplementary Note 1: Manifold Capacity and Effective Geometric Measures

Suppose we have simultaneous measurements of activity across many neurons from an animal presented with two stimulus conditions (e.g., two different odors). Imagine that there is some downstream neuron receiving signals from these recorded neurons: how can it tell the two stimulus conditions apart? Assuming this downstream neuron aggregates information by collecting upstream firing events weighted by the strength of synaptic connectivity, i.e., it essentially uses a weighted sum and then a threshold to determine whether the stimulus is odor A or odor B. Hence, we model such a downstream computation as a linear classification problem.

Before the animal learns to tell the difference between two distinct but similar odors, the two point clouds (or manifolds) of activity patterns could be very tangled with each other in the high-dimensional space where each coordinate corresponds to the activity level of a neuron. Such tangledness makes it hard for a downstream neuron to tell apart these two odors; in other words, it is hard to linearly classify the two odor manifolds. We asked whether the odor manifolds may become untangled and more separated from each other - and, thus, easier to discriminate for a downstream neuron - after the animal was trained in a task that required discrimination between two odors.

To formulate the above intuition into a rigorous quantitative analysis method, we need to answer two questions: (i) how to quantify the degree of separability of two odor manifolds, and (ii) how to connect the properties of the neural activity patterns - such as the firing statistics of neurons, the covariability between neurons, and the structure and shape of odor manifolds - to the degree of separability?

In our analysis, manifold capacity answers question (i) by quantifying the degree of separability of two odor manifolds. Effective geometric measures from GLUE answer question (ii) by rigorously linking the properties of the neural activity patterns to manifold capacity. Now, let's dive into the formal definition of them.

Manifold capacity

Suppose we have multiple datapoints representing simultaneous recordings across a population of neurons (e.g., at different time points or during different trials) from an animal presented with two stimulus conditions (e.g., two different odors). Concretely, let's say we have N neurons recorded, and for each stimulus condition we have M datapoints. Namely, for each stimulus condition, there are M neural activity patterns in the form of N-dimensional vectors where each coordinate corresponds to the activity level of a neuron.

When the number of neurons N is very large ($N \gg M$), these two point clouds (or manifolds) are very likely to be linearly separable from each other due to basic facts from linear algebra. However, when N becomes smaller and smaller (e.g., we randomly sample a subset of neurons), then the chance that the manifolds become linearly inseparable increases. Quantitatively, we can analyze the *manifold separability curve* p(N), defined as the probability of

linear separability after subsampling the number of neurons to N (see Fig. 1a below). The area above the curve corresponds to the critical number of neurons needed for a downstream neuron to read out from for linear classification. We call this number *critical dimension N**.

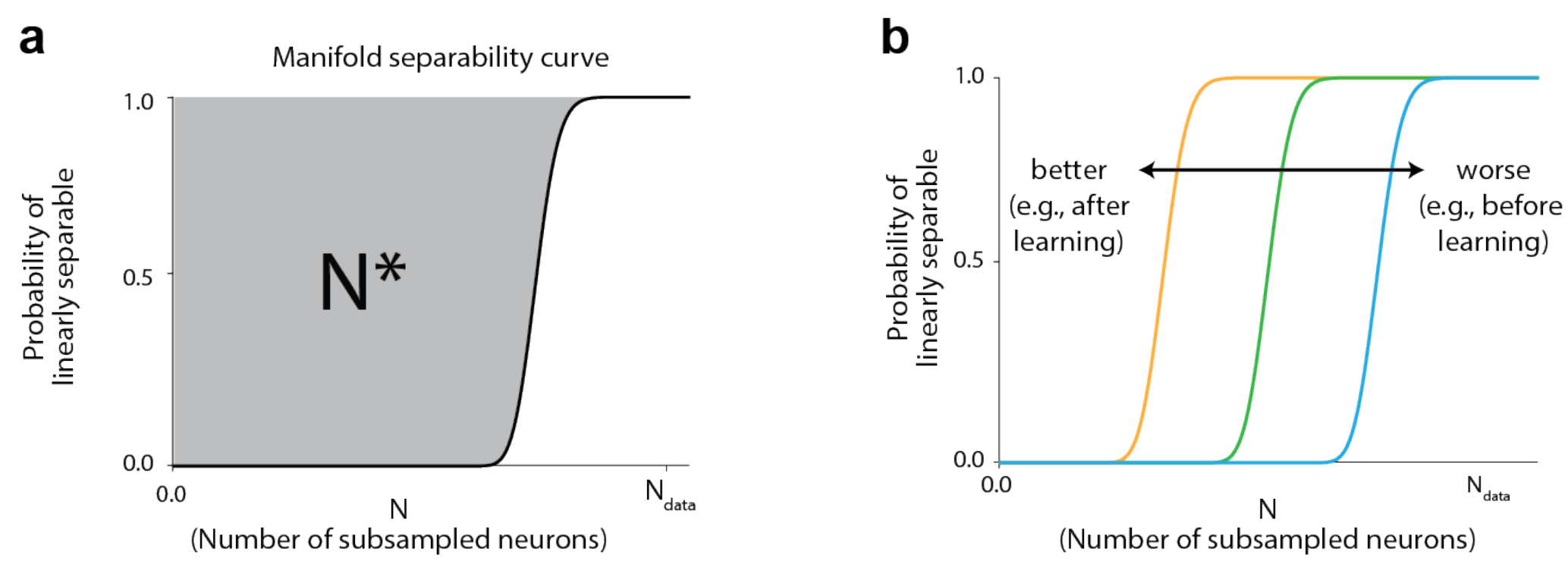

Figure 1: Manifold separability curve. Adapted from ref. 1 (Fig. S2 and S5b).

If the manifold separability curve moves from right to left (see Fig. 1b above), then a downstream neuron can tell the two odors apart using a smaller number of neurons. Namely, the odor manifolds become more separable, and this is quantified by N*. As a result, to evaluate if the separability of odor manifolds improved by training of an animal in a task, we can compare the N* between naïve animals and animals that have been trained. Indeed, what we see in our analysis is that N* is smaller in zebrafish that learned to discriminate the odors.

Manifold capacity α is simply a rescaling of critical dimension by the number of manifolds P (in our case here P = 2): α = P/N*. The larger the capacity is (smaller N*), the more separable the odor manifolds are.

Before moving on to effective geometric measures, we briefly compare manifold capacity to conventional approaches to assess separability. A traditional approach to quantify separability between two manifolds is based on support vector machine (SVM) theory: one identifies the linear classifier with the maximum margin, and the larger this margin, the greater the separability. In contrast, GLUE theory does not focus on the best possible classifier in the full neural space. Instead, it examines linear classifiers built from random sub-populations of neurons. The fewer neurons required for successful separation - the higher the manifold capacity - the more separable the manifolds are. Thus, while SVM captures the best case using all neurons, manifold capacity reflects the average performance across random subsets of neurons.

Finally, we note two key assumptions underlying the theory. First, each object manifold is modeled as the convex hull of its activity patterns. This follows from the fact that, for linear classification, considering the convex hull of points from the same class is equivalent to considering the original points themselves. Second, when analyzing subpopulations of neurons,

we model them as random subsets of the full population. Beyond these, the theory does not impose additional assumptions on the structure of the data. Hence, unlike various alternative measures of separability, GLUE does not approximate manifolds by multivariate normal or other distributions.

Effective geometric measures

The manifold separability curve can be analyzed mathematically, allowing manifold capacity α (and the critical dimension N*) to be estimated directly from a simple formula:

$$\alpha = P \cdot \left(\mathop{\mathbb{E}}_{\substack{\mathbf{y}\sim\mathcal{Y} \\ \mathbf{t}\sim\mathcal{N}(0,I_N)}} \left[\max_{\lambda_i^\mu \geq 0 \ \forall \mu,i} \left(\frac{\langle \mathbf{t}, \sum_{\mu,i} y^\mu \lambda_i^\mu \mathbf{z}_i^\mu \rangle}{\left\| \sum_{\mu,i} y^\mu \lambda_i^\mu \mathbf{z}_i^\mu \right\|_2} \right)^2 \right] \right)^{-1}$$

In the formula above, P is the number of manifolds and $\mathbb{E}$ stands for taking the expectation. y^μ is a class label (y^1 = 1 for odor 1 and y^2 = -1 for odor 2) and the vector $\boldsymbol{t}$ represents a classifier drawn randomly from a multivariate normal distribution (i.e., a random direction in which population activity is projected). The expectation is taken by randomly sampling over y (from a set of label vectors Y) and $\boldsymbol{t}$ (from the isotropic N-dimensional Gaussian distribution $N(0, I_N)$). Inside the expectation is a term that involves maximizing a convex function. The variables $\lambda_i{}^\mu$ are non-negative weights, μ is the index for the manifold, i is the index for the point (i.e., datapoint number) in the μ-th manifold, and $\boldsymbol{z}_i{}^\mu$ is an activity vector (i.e., neural population activity) for the i-th datapoint in the μ-th manifold. Hence, the term $\sum_{\mu,i} y^\mu \lambda_i{}^\mu \boldsymbol{z}_i{}^\mu$ is a convex combination of the activity vectors weighted by the label. Thus, it is also an N-dimensional vector. Finally, in the numerator $\langle\cdot,\cdot\rangle$ stands for the inner product between two vectors, and in the denominator $\|\cdot\|_2$ stands for the L2 norm of a vector. In summary, the capacity formula computes the average (over random draws of y and $\boldsymbol{t}$) of the squared L2 norm of the projection of $\boldsymbol{t}$ onto the convex cone spanned by the activity vectors weighted by their manifold labels. We remark that the random sampling of $\boldsymbol{t}$ is done by random subsampling of neurons; as pointed out above, it does not imply anything about the structure of the data.

This formula has two key implications. First, it enables fast estimation of manifold capacity without explicitly computing the separability curve. Second, it provides an analytical link between manifold capacity and the underlying activity patterns ($\boldsymbol{z}_i{}^\mu$), thereby revealing which structural properties of the manifolds contribute to separability. In what follows, we outline how the GLUE approach leverages this formula to derive effective geometric measures.

1. Anchor point distribution.

When analyzing recordings of neural activity patterns with M datapoints per condition, a common assumption one would make is to treat every datapoint equally. Namely, for each condition, the uniform distribution over the M datapoints would be used to calculate properties such as mean response, covariance, etc.

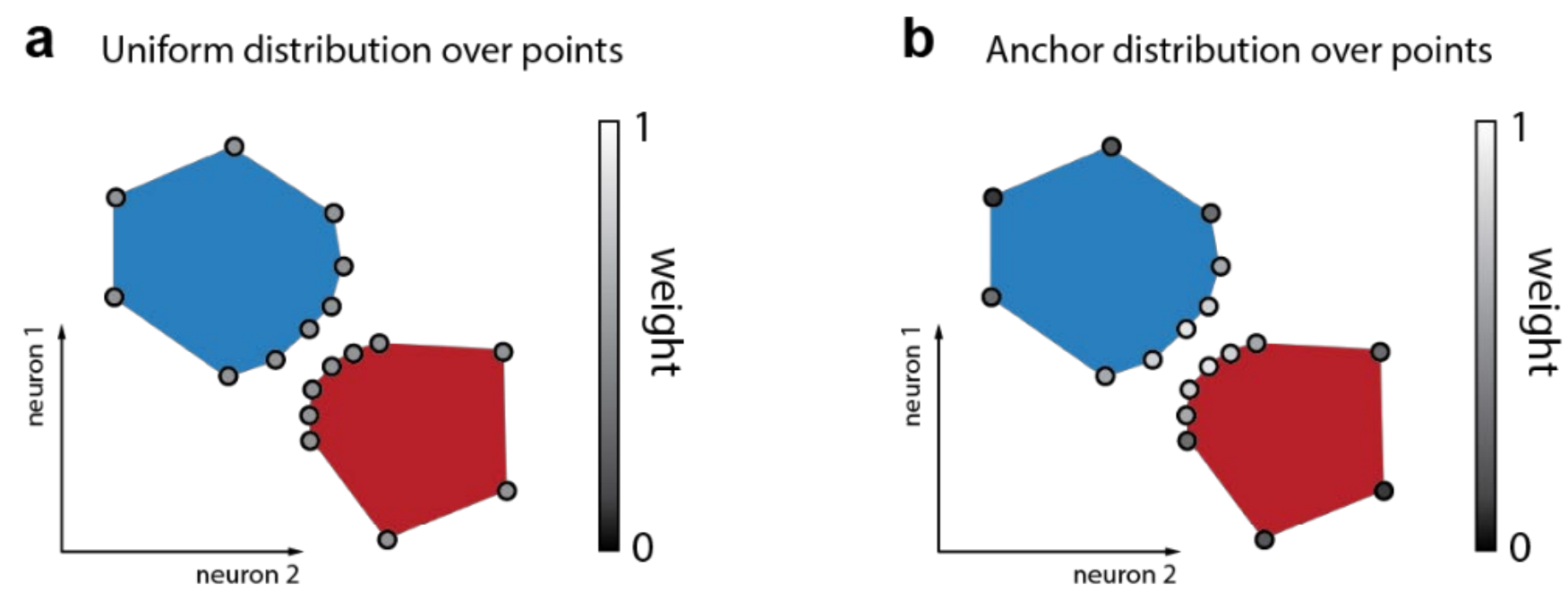

Figure 2: Schematic illustration of the difference between the uniform distribution over data point (**a**) and the anchor point distribution over data points (**b**).

In the GLUE approach, however, we do not draw datapoints randomly from a uniform distribution but choose them based on the manifold capacity formula. Concretely, for each sampled pair of $(y, \boldsymbol{t})$, the optimizer λ_i^{μ} can be thought of as a function of $(y, \boldsymbol{t})$. By rescaling $\lambda_i^{\mu}(y, \boldsymbol{t})$ so that $\sum_i \lambda_i^{\mu}(y, \boldsymbol{t}) = 1$ for each μ, the function $\lambda_i^{\mu}(y, \boldsymbol{t})$ can be interpreted as a probability distribution over the M datapoints from condition μ. This naturally leads to the definition of *anchor points*:

$$\mathbf{s}^{\mu}(\mathbf{y}, \mathbf{t}) := \frac{\sum_i \lambda_i^{\mu}(\mathbf{y}, \mathbf{t}) \mathbf{z}_i^{\mu}}{\sum_i \lambda_i^{\mu}(\mathbf{y}, \mathbf{t})},$$

Intuitively, the anchor with respect to $(y, \boldsymbol{t})$ is the most relevant point in the μ-th manifold for the classification on top of a random sub-population of neurons. Furthermore, in GLUE, the capacity formula can be rewritten as

$$\alpha = P \cdot \left(\mathop{\mathbb{E}}_{\substack{\mathbf{y} \sim \mathcal{Y} \\ \mathbf{t} \sim \mathcal{N}(0, I_N)}} \left[(\mathbf{S}\mathbf{t})^{\top} (\mathbf{S}\mathbf{S}^{\top})^{\dagger} (\mathbf{S}\mathbf{t}) \right] \right)^{-1}$$

Where $\boldsymbol{S}$ is a P × N matrix with $\boldsymbol{s}^{\mu}(y, \boldsymbol{t})$ on its rows. To understand the above variation form of the capacity formula, recall that the previous form of the formula can be understood as the squared L2 norm of the projection of $\boldsymbol{t}$ onto the convex cone spanned by the activity vectors weighted by their manifold labels. Furthermore, the squared L2 norm term can be shown to be the squared L2 norm of the projection of vector $\boldsymbol{t}$ onto the subspace spanned by the anchor points. Through this observation, one can express the projection of $\boldsymbol{t}$ onto this subspace via the orthogonal projection operator $\boldsymbol{S}^{\top}(\boldsymbol{S}\boldsymbol{S}^{\top})^{\dagger}\boldsymbol{S}$. $\boldsymbol{S}\boldsymbol{t}$ is the projection of the random vector $\boldsymbol{t}$ onto the anchor points, $\boldsymbol{S}\boldsymbol{S}^{\top}$is the covariance (Gram) matrix of the anchor points (representing the "geometry" of the anchor space), and † denotes the pseudoinverse. The projection operator

therefore quantifies the squared length of the projection of $\boldsymbol{t}$ onto the anchor points, measured in the space spanned by the anchor points after correcting for their covariances.

To summarize, the manifold capacity is now analytically connected to a simple formula (i.e., the squared L2 norm of the projection of vector $\boldsymbol{t}$ to the subspace spanned by the anchor points, averaged with respect to the anchor point distribution).

2. Center-axis decomposition.

Next, after replacing the uniform distribution over the datapoints from the same experimental condition with the anchor point distribution, we can conduct standard statistical analyses - such as estimating mean and variance of the responses - by averaging with respect to the anchor point distribution as opposed to the conventionally used uniform distribution. Conventionally, one would have defined the center of the μ-th manifold as the average of the activity patterns over all datapoints, i.e., $\frac{1}{M}\sum_i \boldsymbol{z}_i^{\mu}$. Now, it is natural to define the center of the μ-th manifold as the average of its anchor points, i.e., $\boldsymbol{s}_0^{\mu} = E_{y,\boldsymbol{t}}\,[\boldsymbol{s}^{\mu}(y,\boldsymbol{t})]$. Furthermore, the axis part of an anchor point is then defined as $\boldsymbol{s}_1^{\mu}(y,\boldsymbol{t}) = \boldsymbol{s}^{\mu}(y,\boldsymbol{t}) - \boldsymbol{s}_0^{\mu}$.

Intuitively, the above *anchor center-axis decomposition* is analogous to what one would conventionally do by segregating the set of datapoints into components representing their mean and their variation. The main difference is that, traditionally, the mean is defined as the uniform average over the datapoints, while here the average is calculated with respect to the anchor point distribution. Likewise, the variation is determined here based on the anchor point distribution rather than the uniform distribution.

3. Effective manifold dimension and radius.

After segregating the center and axis components of an anchor point we define *effective geometric measures* such as effective dimension and effective radius that are (i) analogous to the conventional measures for manifold dimension and radius using the uniform distribution and (ii) analytically linked to the capacity formula. In particular, in the original GLUE paper[1], the authors showed that these effective geometric measures can faithfully track the ground truth dimension and radius of simulated examples when the underlying correlations between manifolds are mild. And when the correlations are strong, the effective geometric measures can more robustly track their effect on classification efficiency.

<u>Formulas for effective manifold dimension and radius:</u>

Similar to the matrix $\boldsymbol{S}$ defined above, here we define matrix $\boldsymbol{S}_0$ and $\boldsymbol{S}_1$ as P × N matrices with $\boldsymbol{s}_0^{\mu}$ and $\boldsymbol{s}_1^{\mu}(y,\boldsymbol{t})$ on their rows, respectively. In GLUE, the following three quantities were defined:

$$
\begin{aligned}
a(\mathbf{y},\mathbf{t}) &= (\mathbf{S}\mathbf{t})^{\top}(\mathbf{S}\mathbf{S}^{\top})^{\dagger}(\mathbf{S}\mathbf{t}),\\
b(\mathbf{y},\mathbf{t}) &= (\mathbf{S}_1(\mathbf{y},\mathbf{t})\mathbf{t})^{\top}\left(\mathbf{S}_1(\mathbf{y},\mathbf{t})\mathbf{S}_1(\mathbf{y},\mathbf{t})^{\top}\right)^{\dagger}(\mathbf{S}_1(\mathbf{y},\mathbf{t})\mathbf{t}),\\
c(\mathbf{y},\mathbf{t}) &= (\mathbf{S}_1(\mathbf{y},\mathbf{t})\mathbf{t})^{\top}\left(\mathbf{S}_0\mathbf{S}_0^{\top} + \mathbf{S}_1(\mathbf{y},\mathbf{t})\mathbf{S}_1(\mathbf{y},\mathbf{t})^{\top}\right)^{\dagger}(\mathbf{S}_1(\mathbf{y},\mathbf{t})\mathbf{t}).
\end{aligned}
$$

Here $a(\boldsymbol{y}, \boldsymbol{t})$ is the squared L2 norm of the projection of vector $\boldsymbol{t}$ onto the subspace spanned by the anchor points as we saw in the capacity formula. $b(\boldsymbol{y}, \boldsymbol{t})$ is an analogous projection term but replacing anchor points with their axial parts. $c(\boldsymbol{y}, \boldsymbol{t})$ is a correction term between $a(\boldsymbol{y}, \boldsymbol{t})$ and $b(\boldsymbol{y}, \boldsymbol{t})$ assuming no center-axis correlations (i.e., removing all cross terms that contain inner products (covariances) between the center part of an anchor point and the axis part of an anchor point).

Notice that the capacity $\alpha = P/E_{\boldsymbol{y},\boldsymbol{t}}[a(\boldsymbol{y}, \boldsymbol{t})]$. Now, the effective dimension and radius are defined as

$$D_{\text{eff}} := \frac{1}{P}\,\mathbb{E}_{\mathbf{y},\mathbf{t}}[b(\mathbf{y},\mathbf{t})]$$

and

$$R_{\text{eff}} := \sqrt{\frac{\mathbb{E}_{\mathbf{y},\mathbf{t}}[c(\mathbf{y},\mathbf{t})]}{\mathbb{E}_{\mathbf{y},\mathbf{t}}[b(\mathbf{y},\mathbf{t}) - c(\mathbf{y},\mathbf{t})]}}$$

To build up intuitions for effective dimension and radius, it is helpful to first consider a scenario where there is no correlation between the anchor points across manifolds. Namely, the inner product between $\boldsymbol{s}^{\mu}(y, \boldsymbol{t})$ and $\boldsymbol{s}^{\nu}(y, \boldsymbol{t})$ is zero when $\mu \neq \nu$. In this case, the above equations can be simplified:

$$D_{\text{eff}} \approx \frac{1}{P}\sum_{\mu} \mathop{\mathbb{E}}_{\mathbf{y},\mathbf{t}}\left[\frac{(\langle \mathbf{s}_1^{\mu}(\mathbf{y},\mathbf{t}), \mathbf{t}\rangle)^2}{\|\mathbf{s}_1^{\mu}(\mathbf{y},\mathbf{t})\|^2}\right]$$

and

$$R_{\text{eff}}^2 \approx \frac{1}{P}\sum_{\mu} \frac{\mathbb{E}_{\mathbf{y},\mathbf{t}}[\|\mathbf{s}_1^{\mu}(\mathbf{y},\mathbf{t})\|^2]}{\|\mathbf{s}_0^{\mu}\|^2}$$

Now, both effective dimension and radius can be approximated by an average of some quantities about each individual manifold. Furthermore, if the manifolds are D-dimensional balls with radius (normalized by the length of the manifold center) being R, then the quantity on the right-hand side of the equations are exactly D and R^2, respectively.

When there are correlations between manifolds, the effective dimension and radius can detect their effect on the separability (through the analytical connection to manifold capacity). Specifically, the GLUE approach shows that the effective dimension captures the correlation between manifolds' axes (i.e., within-manifold variations) while the effective radius captures the correlations between manifolds' centers (i.e., mean responses). See Figure S6 in GLUE paper[1] for a quantitative example.

4. Anchor statistics.
Finally, one can also utilize simple statistics from the anchor points to quantify structure in data, just like what is conventionally done by using mean firing rate and/or covariance etc. Some common statistical measures from anchor point distribution include:

- Anchor center alignment: For manifold μ and manifold ν, the anchor center alignment between the two is:

$$\rho^c_{\mu,\nu} := |\langle \mathbf{s}_0^\mu, \mathbf{s}_0^\nu \rangle|$$

  which captures the cosine similarity between the mean responses defined by weighting over the anchor points (as opposed to over the uniform distribution).
- Anchor axis alignment: For manifold μ and manifold ν, the anchor axis alignment between the two is:

$$\rho^a_{\mu,\nu} := \mathop{\mathbb{E}}_{\mathbf{y},\mathbf{t}}[|\langle \mathbf{s}_1^\mu(\mathbf{y},\mathbf{t}), \mathbf{s}_1^\nu(\mathbf{y},\mathbf{t}) \rangle|]$$

  which captures the cosine similarity between the within-manifold variations defined by weighting over the anchor points (as opposed to over the uniform distribution).
- Anchor center-axis alignment: For manifold μ and manifold ν, the anchor center alignment between the two is:

$$\psi_{\mu,\nu} := \mathop{\mathbb{E}}_{\mathbf{y},\mathbf{t}}[|\langle \mathbf{s}_0^\mu, \mathbf{s}_1^\nu(\mathbf{y},\mathbf{t}) \rangle|]$$

  which captures the cosine similarity between the mean responses and within-manifold variations defined by weighting over the anchor points (as opposed to over the uniform distribution).

In all our analysis, we consider the average anchor statistical measures by taking the mean over all pairs of manifolds.

Summary of effective geometric measures.
To summarize, the GLUE theory establishes an analytical connection between separability and manifold geometry through a formula for manifold capacity. This formula naturally defines a non-uniform distribution over points on the manifolds, referred to as the anchor point distribution. Building on this, the theory introduces two classes of geometric measures:

1. **Effective dimension and effective radius**, which reduce to the conventional notions of dimension and radius when correlations are weak but also incorporate corrections that account for correlations relevant to downstream classification.

2. **Anchor statistics** - including center, axis, and center–axis alignment - which parallel conventional statistical measures but are computed with respect to the anchor point distribution rather than a uniform distribution, thereby capturing task-relevant structural properties of the manifolds.

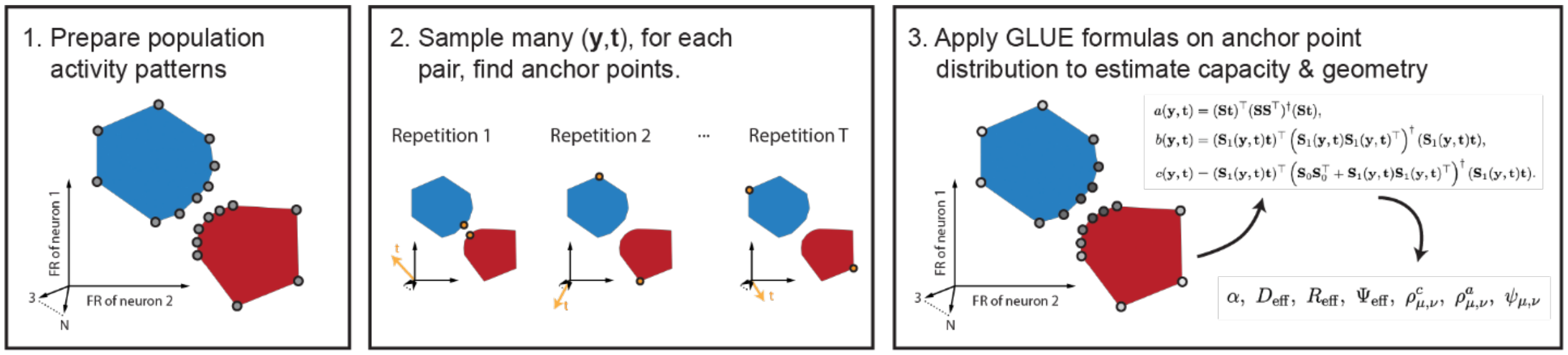

Figure 3: Schematic: Estimation of GLUE geometry from the anchor point distribution.

Difference Between GLUE Analysis & Conventional Methods

GLUE analysis provides two types of measurements for data analysis: (i) manifold capacity, which quantifies the degree of manifold separability, and (ii) task-relevant geometric measures, which quantify the contributions of structural properties of the manifolds to their separability. The key novelty of GLUE is that it establishes a rigorous mathematical link between these two levels of measurement, thereby connecting function (separability) and structure (geometry). Importantly, unlike many conventional analysis methods that rely on strong distributional assumptions (e.g., normality or Gaussian noise models), GLUE makes no such assumptions about the underlying data, making it broadly applicable to high-dimensional, complex neural representations.

To provide more intuition for GLUE analysis, we include below a comparison with conventional data analysis methods. Specifically, we compare GLUE analysis with (a) decoding analysis, (b) dimensionality reduction analysis (e.g., PCA, UMAP), and (c) spectral analysis (e.g., participation ratio).

Comparison between GLUE and decoding analysis

A common approach in neural data analysis is decoding with linear classifiers. The most basic example is *Linear Discriminant Analysis (LDA)*, which assumes that neural responses for each class follow Gaussian distributions with shared covariance. LDA assesses separability by finding the projection that maximizes the ratio of between-class to within-class variance. While simple and interpretable, this approach depends strongly on normality assumptions and covariance estimation, which are often violated in real neural data.

An advanced alternative to LDA is the *Support Vector Machine (SVM)*, which does not rely on distributional assumptions and instead finds the separating hyperplane that maximizes the margin between classes. SVM provides a “best-case” estimate of separability given the full set of recorded neurons.

In contrast, GLUE is not tied to any particular classifier and makes no assumptions about data normality. Rather than evaluating separability once with a single optimal decision boundary, GLUE quantifies how reliably random subsets of neurons can achieve linear classification. The

fewer neurons required for reliable separation, the higher the manifold capacity. This shift provides a population-level, assumption-free, and geometrically interpretable measure of separability that links *function* (classification capacity) with *structure* (manifold geometry).

Comparison between GLUE and dimensionality reduction

Dimensionality reduction methods such as PCA or UMAP summarize neural activity by projecting it into a lower-dimensional space, inevitably discarding part of the original information. These projections are often useful for visualization or exploratory analysis but the resulting coordinates are not directly tied to variables of interest such as task performance. In contrast, GLUE defines its geometric measures - such as effective dimension, radius, and anchor statistics - directly in the original high-dimensional activity space. Rather than compressing the data, GLUE extracts task-relevant geometric structure without information loss from projection, ensuring that the measures remain fully connected to manifold separability.

Comparison between GLUE and spectral analysis

Spectral analysis methods study the structure of neural activity by looking at the eigenvalue spectrum of the covariance matrix. For example, the participation ratio is a common metric that estimates the dimensionality of neural responses based on how variance is spread across eigenmodes. These measures provide a global picture of variability but rely on covariances computed with uniform weighting over all data points. In contrast, GLUE defines its effective geometric measures and anchor statistics using the anchor point distribution, which emerges directly from the manifold capacity formula. Because this distribution is task-relevant, the resulting measures are also task-relevant: they describe the geometry and statistics of manifolds in direct relation to their separability.

Computational interpretation of manifold capacity: additional remarks

Unlike classical approaches, GLUE does not analyze optimal linear classification based on all neurons but asks how many randomly selected neurons are, on average, required to classify manifolds with a given probability. This approach more directly reflects the readout of information in biological networks where decoding neurons typically do not have access to all neurons in a network. Moreover, the resulting measure of separability - manifold capacity - differs from classical measures with respect to its computational interpretation. Manifold capacity is directly and analytically linked to computationally relevant quantities (see ref. 1 for mathematical description):

(1) *Efficiency* (how many neurons are needed for the retrieval of information?). Higher manifold capacity means that the retrieval of information contained in neural manifolds requires access to fewer neurons in the network. Hence, less resources are required for readout. Moreover, finding a classifier (the learning process) becomes easier.

(2) *Packing density* (how many manifolds can be fit into the activity space?). Higher manifold capacity means that more manifolds, each representing different information, can be packed into activity space, implying higher storage capacity.

(3) *Robustness* (how much imperfection can be tolerated in the classifier?). Higher manifold capacity means that performance degrades more slowly when classifiers deviate from the optimum, implying that classification becomes more noise-resistant. Moreover, as it becomes easier to find classifiers with suboptimal yet acceptable performance, learning processes are facilitated and accelerated.

In summary, classical approaches such as SVMs lack biological plausibility and provide limited information on relevant features of neural representations such as efficiency and robustness because they focus on classification under optimal conditions. Hence, manifold capacity is a novel measure that provides additional computationally relevant information. From a neuroscience perspective, manifold capacity is of particular interest because it can be regarded as a measure that quantifies the "suitability" of neural manifolds for the representation of task-relevant information in biological (and artificial) networks.

A Simulated Toy Example for Intuition Building

Here we provide a toy example of synthetic Gaussian clouds, simulating representational odor manifolds, and examine how manifold capacity and geometric measures change with respect to the latent parameters in the simulated model. These examples were modified from the supplementary materials of the GLUE paper[1].

Concretely, we consider $P = 2$ manifolds, each containing M points in an N-dimensional neural activity space. In all examples below we pick $M = 200$ and $N = 1000$, and we remark that for other choices of M and N the results would hold qualitatively. There are 4 parameters to determine the structure and correlations of manifolds:

- d: latent manifold dimension; we consider values ranging from 2 to 10.
- r: latent manifold radius; we consider values ranging from 0.3 to 2.
- σ_c^2: latent center correlations; we consider values ranging from 0 to 1.
- σ_a^2: latent axis correlations; we consider values ranging from 0 to 1.

The following is the procedure to generate two synthetic Gaussian clouds from the above parameters:

1. Randomly sample $\boldsymbol{u}_0{}^1$, $\boldsymbol{u}_0{}^2$ independently from N-dimensional isotropic Gaussian distribution with mean 0 and variance $1/\sqrt{N}$ in each coordinate.
2. Correlate each pair of coordinates from $\boldsymbol{u}_0{}^1$, $\boldsymbol{u}_0{}^2$ so that the covariance becomes σ_c^2.
3. Randomly sample $\boldsymbol{u}_1{}^1, \ldots, \boldsymbol{u}_d{}^1$ and $\boldsymbol{u}_1{}^2, \ldots, \boldsymbol{u}_d{}^2$ independently from N-dimensional isotropic Gaussian distribution with mean 0 and variance $1/\sqrt{N}$ in each coordinate.
4. For each $i = 1, \ldots, d$, correlate each pair of coordinates from $\boldsymbol{u}_i{}^1$, $\boldsymbol{u}_i{}^2$ so that the covariance becomes σ_a^2.
5. Repeat the following for M times for each manifold (indexed by μ): sample $(b_1, b_2, \ldots, b_d)$ from the d-dimensional unit sphere, and output $\boldsymbol{u}_0{}^\mu + \sum_{i=1}^{d} r b_i \boldsymbol{u}_i{}^\mu$.

Recall that GLUE analysis gives us the following measures: manifold capacity α, effective manifold dimension D_{eff}, effective manifold radius R_{eff}, anchor center alignment ρ_c, anchor axis alignment ρ_a, and anchor center-axis alignment ψ. In the following, we are going to see how different values of the latent parameters (d, r, σ_c^2, σ_a^2) in the simulated model mapped to the GLUE measures (α, D_{eff}, R_{eff}, ρ_c, ρ_a, ψ).

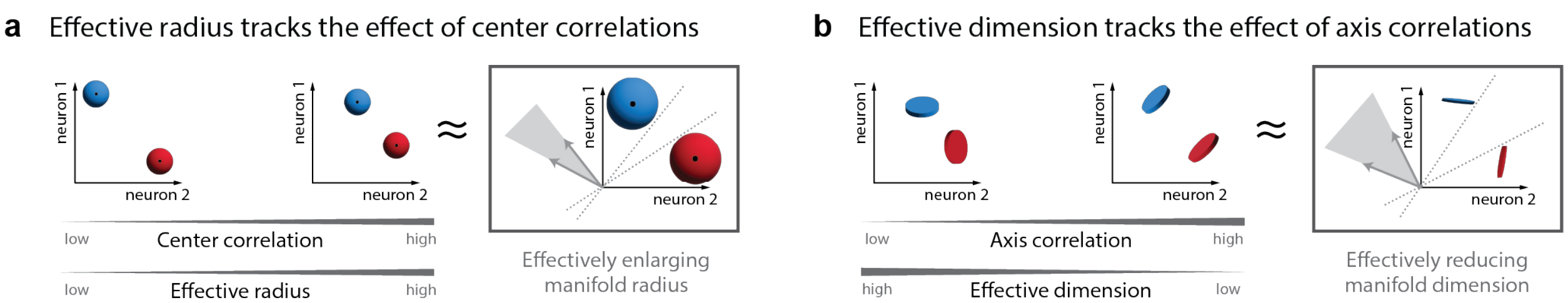

Figure 4: Schematic: connection between latent geometry and GLUE effective radius (**a**) and effective dimension (**b**).

Case 1: No correlation between manifolds

Let us start with simple cases where $\sigma_c^2 = \sigma_a^2 = 0$. We swept through a grid of latent parameters with d in [2, 10] and r in [0.1, 2]. For each pair of (d, r), we ran 50 repetitions of random simulations and analyzed the resulting GLUE measures. To understand the results, first we plot all GLUE measures with respect to varying the latent manifold radius r as follows.

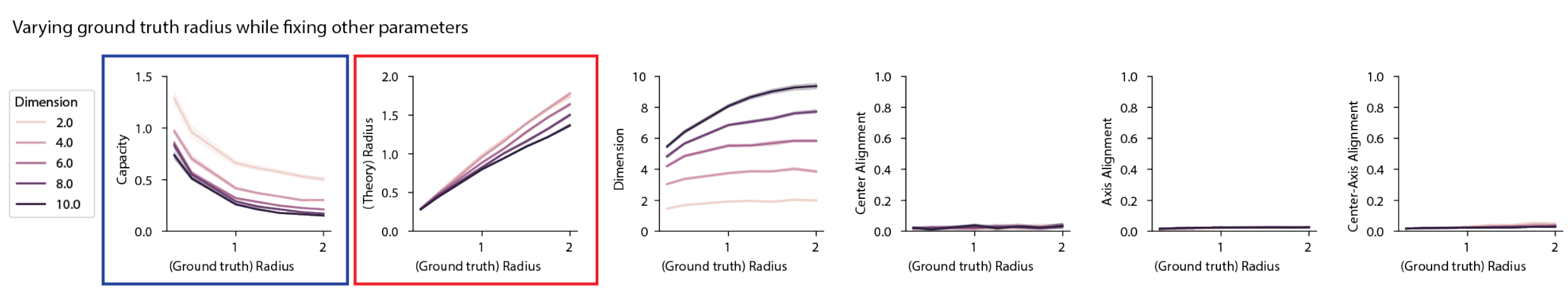

Notice that manifold capacity α decreases as the latent manifold radius r increases, agreeing with the intuition that manifolds become more tangled when r is larger. Moreover, the effective manifold radius R_{eff} faithfully tracks the value of latent manifold radius r, especially when the latent manifold dimension d is small (remark: the reason for the decrease in R_{eff} when d becomes large is a finite size effect: because M is small, the simulated procedure cannot sample the whole sphere when d is large).

Similarly, we plot all GLUE measures with respect to varying the latent manifold dimension d as follows.

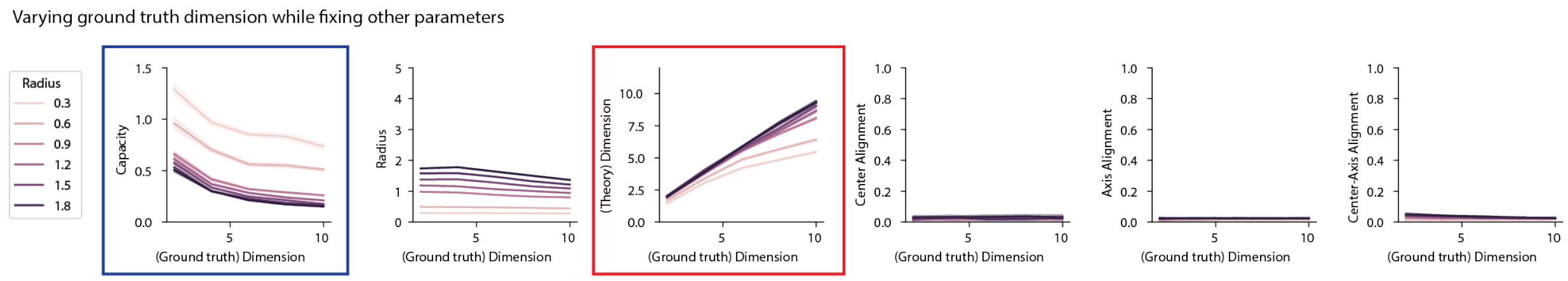

Notice that manifold capacity α decreases as the latent manifold radius d increases, agreeing with the intuition that manifolds become more tangled when d is larger. Moreover, the effective manifold dimension D_{eff} faithfully tracks the value of latent manifold dimension d, especially when the latent manifold dimension r is large (remark: the reason why D_{eff} decreases when r becomes small is that the point clouds become more point-like when the radius is small).

We conclude that in the regime when there are no correlations between the manifolds, effective dimension and radius (D_{eff}, R_{eff}) faithfully track the value of latent dimension and radius (d, r).

Case 2: Introducing correlation between manifold centers

Next, we investigate the effect of correlation between manifold centers on capacity and GLUE geometry. We set $d = 4$, $r = 1$, and swept through a grid of latent parameters with σ_c^2, σ_a^2 in [0, 1]. For each pair of (σ_c^2, σ_a^2), we ran 50 repetitions of random simulations and analyzed the resulting GLUE measures. To understand the results, first we plot all GLUE measures with respect to varying the latent manifold center correlations σ_c^2 as follows.

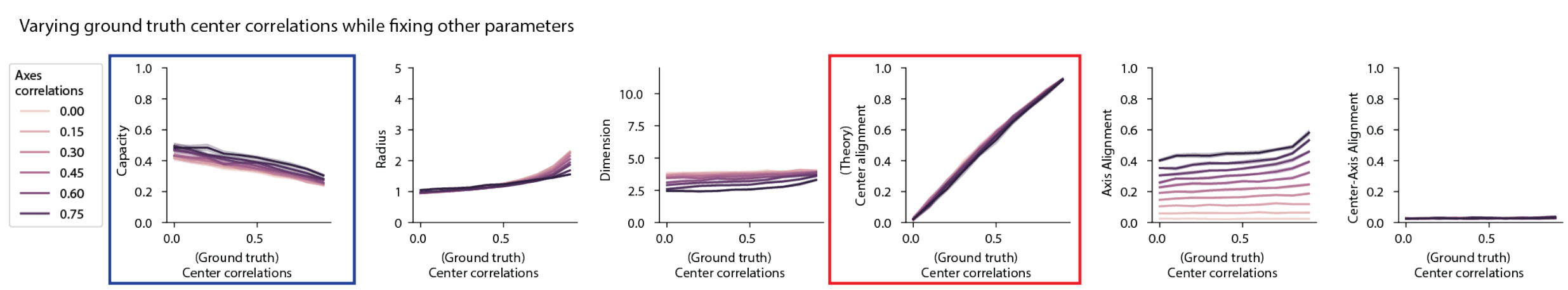

Notice that manifold capacity α decreases as the latent manifold center correlations σ_c^2 increase, agreeing with the intuition that manifolds become more tangled when they are closer to each other. Moreover, the anchor center alignment ρ_c faithfully tracks the value of latent manifold center correlations σ_c^2. In addition, effective radius R_{eff} also tracks the effect of center correlations (as explained intuitively in Fig. 4a). Specifically, the larger the latent manifold center correlations σ_c^2, effectively the size of the manifolds becomes larger (hence decreasing capacity).

Case 3: Introducing correlation between manifold axes

Finally, we investigate the effect of correlation between manifold axes on capacity and GLUE geometry. We set $d = 4$, $r = 1$, and swept through a grid of latent parameters with σ_c^2, σ_a^2 in [0, 1]. For each pair of (σ_c^2, σ_a^2), we ran 50 repetitions of random simulations and analyzed the resulting GLUE measures. To understand the results, first we plot all GLUE measures with respect to varying the latent manifold axis correlations σ_a^2 as follows.

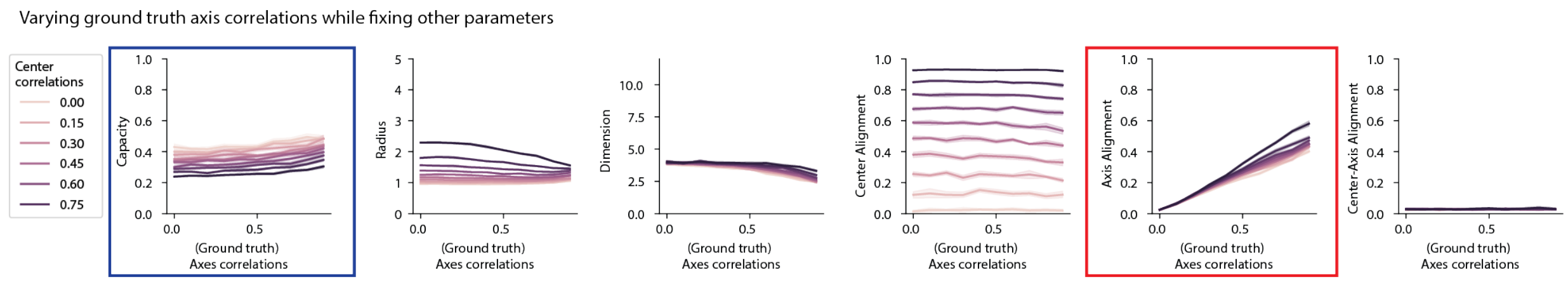

Notice that manifold capacity α increases as the latent manifold axis correlations σ_a^2 increase, agreeing with the intuition that manifolds become more untangled when they are internally more aligned with each other. Moreover, the anchor axis alignment ρ_a faithfully tracks the latent manifold axis correlations σ_a^2. In addition, effective dimension D_{eff} also tracks the effect of axis correlations (as explained intuitively in Fig. 4b). Specifically, as latent manifold axis correlations σ_a^2 increase, effectively the manifolds become lower dimensional (hence increasing capacity).

References

1. Chou C-N, Kim R, Arend LA, Yang Y-Y, Mensh BD, Shim WM, Perich MG, Chung S (2024) Neural Manifold Capacity Captures Representation Geometry, Correlations, and Task-Efficiency Across Species and Behaviors. bioRxiv:2024.2002.2026.582157; doi: https://doi.org/10.1101/2024.02.26.582157

SUPPLEMENTARY NOTE 2 | Detailed information on statistical analyses shown in figures

Fig. 1f: Appetitive ζ scores were significantly higher for CS+ than CS- on all days (day 1, N = 25 fish, $P = 6.0 \times 10^{-8}$; day 2, N = 25, $P = 1.8 \times 10^{-7}$; day 3, N = 21, $P = 6.7 \times 10^{-6}$; day 4, N = 14, $P = 1.2 \times 10^{-4}$; two-tailed Wilcoxon signed-rank test). P-values reported in the legend were not adjusted for multiple comparisons.

Fig. 1h: Activity was significantly higher in all trained groups (Arg^+/Phe^-: 0.95 ± 1.06 [mean ± SD], n_{odor} = 3, n = 13013 neurons from N = 9 fish; Phe^+/Arg^-: 1.07 ± 1.10, n_{odor} = 3, n = 13379 neurons from N = 10 fish); Phe^+/Trp^-: 1.03 ± 1.11, n_{odor} = 3, n = 7760 neurons from N = 6 fish) than in naïve fish (0.90 ± 0.93, n_{odor} = 3, n = 9059 neurons from N = 6 fish; Kruskal–Wallis test, n = 129633, $P = 3.7 \times 10^{-143}$). Posthoc Dunn's test: Arg^+/Phe^- vs naïve, $P = 2.9 \times 10^{-10}$; Phe^+/Arg^- vs naïve, $P = 6.1 \times 10^{-104}$; Phe^+/Trp^- vs naïve, $P = 2.8 \times 10^{-45}$. P-values were adjusted for multiple comparisons by Bonferroni correction.

Fig. 2b: Amino acids vs amino acids (AA-AA), naïve (mean ± SD): 0.336 ± 0.064, n = 18 odor pairs from N = 6 fish; trained: 0.354 ± 0.053, n = 75 odor pairs from N = 25 fish, Mann–Whitney U test, P = 0.107. Amino acids vs bile acids (AA-BA), naïve: 0.433 ± 0.058, n = 54 odor pairs from N = 6 fish; trained: 0.471 ± 0.057, n = 225 odor pairs from N = 25 fish, Mann–Whitney U test, P = 0.0002. Bile acids vs bile acids (BA-BA), naïve: 0.354 ± 0.055, n = 18 odor pairs from N = 6 fish; trained: 0.389 ± 0.054, n = 75 odor pairs from N = 25 fish, Mann–Whitney U test, P = 0.0007. P-values were not adjusted for multiple comparisons.

Fig. 2c: Amino acids, naïve (mean ± SD): 0.270 ± 0.076, n = 54 trial pairs from N = 6 fish; trained: 0.257 ± 0.069, n = 225 trial pairs from N = 25 fish, Mann–Whitney U test, P = 0.202. Bile acids, naïve: 0.318 ± 0.060, n = 54 trial pairs from N = 6 fish; trained: 0.329 ± 0.076, n = 225 trial pairs from N = 25 fish, Mann–Whitney U test, P = 0.264). P-values were not adjusted for multiple comparisons.

Fig. 2e: LDA cross-validation accuracy was significantly higher in all trained groups relative to the naïve group across all sample sizes. For 200 neurons, group differences were significant (Kruskal–Wallis test, n = 465 odor pairs from N = 31 fish, $P = 3.7 \times 10^{-9}$), with post-hoc Dunn tests showing significant differences between the naïve group and Arg^+/Phe^- ($P = 7.0 \times 10^{-8}$), Phe^+/Arg^- ($P = 2.5 \times 10^{-6}$), and Phe^+/Trp^- (P = 0.018). For sample sizes of 300, 400, 500, 600, and 700 neurons, group differences remained significant (Kruskal–Wallis tests: $P = 1.8 \times 10^{-8}$, 1.8×10^{-7}, 4.0×10^{-7}, 1.0×10^{-6}, and 4.9×10^{-6}, respectively), and post-hoc comparisons against the naïve group were significant for Arg^+/Phe^- ($P = 3.1 \times 10^{-7}$, 4.7×10^{-6}, 2.0×10^{-5}, 8.6×10^{-5}, and 4.0×10^{-4}), Phe^+/Arg^- ($P = 4.5 \times 10^{-6}$, 1.3×10^{-5}, 3.5×10^{-5}, 8.8×10^{-5}, and 5.0×10^{-4}), and Phe^+/Trp^- (P = 0.009, 0.008, 0.010, 0.008, and 0.015), respectively. P-values were adjusted for multiple comparisons by Bonferroni correction.

Fig. 4e: dE between manifolds representing two amino acids (AA↔AA), and between manifolds representing one amino acid and one bile acid (AA↔BA), was significantly higher in trained fish (AA↔AA: 8.05 ± 2.27, mean ± SD; n = 75 pairs from N = 25 fish; AA↔BA: 8.89 ± 2.50, n = 225 pairs from N = 25 fish) than in naïve fish (AA↔AA: 6.53 ± 1.26, n = 18 pairs from N = 6 fish, Mann–Whitney U test, P = 0.009; AA↔BA: 7.51 ± 1.76, n = 54 pairs from N = 6 fish, P = 0.0003). dE between manifolds representing two bile acids (BA↔BA) was not significantly different between trained (5.22 ± 1.42, n = 75 pairs from N = 25 fish) and naïve fish (4.93 ± 0.61, n = 18 pairs from N = 6 fish, Mann–Whitney U test, P = 0.253). All statistical tests were two-sided. P-values were not adjusted for multiple comparisons..

Fig. 4f: dM was significantly higher in trained fish between manifolds representing amino acids (AA→AA; trained: 341.3 ± 90.8, n = 150 pairs from N = 25 fish; naïve: 308.6 ± 67.4, n = 36 pairs from N = 6 fish, Mann–Whitney U test, $P = 0.044$) and in the direction from amino acids to bile acids (AA→BA; trained: 492.0 ± 118.1, n = 225 pairs from N = 25 fish; naïve: 414.3 ± 98.6, n = 54 pairs from N = 6 fish, Mann–Whitney U test, $P = 3.1 \times 10^{-6}$). No significant differences in dM between trained and naïve fish were observed in the direction from bile acids to amino acids (BA→AA; trained: 273.5 ± 53.0, n = 225 pairs from N = 25 fish; naïve: 280.3 ± 40.6, n = 54 pairs from N = 6 fish, Mann–Whitney U test, $P = 0.657$) and between manifolds representing bile acids (BA→BA; trained: 271.7 ± 50.4, n = 150 pairs from N = 25 fish; naïve: 264.3 ± 24.5, n = 36 pairs from N = 6 fish, Mann–Whitney U test, $P = 0.272$). All statistical tests were two-sided. P-values were not adjusted for multiple comparisons..

Fig. 5b: Left: Naïve (mean ± SD): 0.100 ± 0.011, n = 90 odor pairs from N = 6 fish; trained: 0.108 ± 0.014, n = 375 odor pairs from N = 25 fish (Mann–Whitney U test, $P = 2.8 \times 10^{-7}$). Shuffled naïve: 0.014 ± 0.0001; shuffled trained: 0.014 ± 0.0001 (Mann–Whitney U test, $P = 0.851$). Right: Manifold capacity was significantly increased in all trained groups relative to the naïve group. Arg^+/Phe^- (mean ± SD): 0.112 ± 0.019, n = 135 odor pairs from N = 9 fish; Phe^+/Arg^-: 0.106 ± 0.010, n = 150 odor pairs from N = 10 fish; Phe^+/Trp^-: 0.105 ± 0.010, n = 90 odor pairs from N = 6 fish. Differences in manifold capacity were statistically significant between the naïve group (0.100 ± 0.011, n = 90 odor pairs from N = 6 fish) and each of the three training groups (Kruskal–Wallis test, n = 465, d.f. = 3, $H = 31.08$, $P = 8.2 \times 10^{-7}$; post-hoc Dunn test: Arg^+/Phe^-, $P = 1.2 \times 10^{-7}$; Phe^+/Arg^-, $P = 5.6 \times 10^{-5}$; Phe+/Trp^-, $P = 0.002$). Gray: manifold capacity after shuffling manifold labels was not significantly different between the naïve and trained groups (Kruskal–Wallis test, $P = 0.965$). All statistical tests were two-sided. P-values were adjusted for multiple comparisons by Bonferroni correction.

Fig. 5c: Amino acids versus amino acids (AA-AA; mean ± SD), naïve: 0.094 ± 0.006, n = 18 pairs from N = 6 fish; trained: 0.103 ± 0.010, n = 75 pairs from N = 25 fish, Mann–Whitney U test, $P = 7.4 \times 10^{-5}$. Amino acids versus bile acids (AA-BA), naïve: 0.105 ± 0.011, n = 54 pairs from N = 6 fish; trained: 0.114 ± 0.013, n = 225 pairs from N = 25 fish, Mann–Whitney U test, $P = 1.7 \times 10^{-5}$. Bile acids versus bile acids (BA-BA), naïve: 0.089 ± 0.005, n = 18 pairs from N = 6 fish; trained: 0.094 ± 0.008, n = 75 pairs from N = 25 fish, Mann–Whitney U test, $P = 0.009$. All statistical tests were two-sided. P-values were not adjusted for multiple comparisons..

Fig. 5e: Effective radius in the trained group (mean ± SD 0.953 ± 0.041, n = 375 pairs from N = 25 fish) was significantly lower than in the naïve group (0.986 ± 0.039, n = 90 pairs from N = 6 fish; Mann–Whitney U test, $P = 4.0 \times 10^{-10}$). Effective dimension in the trained group (17.0 ± 1.9) was significantly lower than in the naïve group (17.7 ± 1.6; Mann–Whitney U test, $P = 0.0007$). Effective center alignment in the trained group (0.808 ± 0.049) was significantly lower than in the naïve group (0.835 ± 0.044; Mann–Whitney U test, $P = 5.0 \times 10^{-5}$). Effective axes alignment in the trained group (0.099 ± 0.028) was significantly lower than in the naïve group (0.109 ± 0.028; Mann–Whitney U test, $P = 0.006$). All statistical tests were two-sided. P-values were adjusted for multiple comparisons by Bonferroni correction.

Extended Data Fig. 2d: Cross-validation accuracy of pairwise odor classification by a support vector machine was significantly higher in trained groups than in the naïve group across all sample sizes. For 200 neurons, group differences were significant (Kruskal–Wallis test, n = 465 odor pairs from N = 31 fish, $P = 2.9 \times 10^{-9}$), with post-hoc Dunn tests showing significant differences between the naïve group and

Arg^+/Phe^- (P = 1.5 × 10^{-6}), Phe^+/Arg^- (P = 4.0 × 10^{-7}), but not Phe^+/Trp^- (P = 0.264). For sample sizes of 300, 400, 500, 600, and 700 neurons, group differences remained significant (Kruskal–Wallis tests: P = 4.6 × 10^{-9}, 1.6 × 10^{-8}, 1.5 × 10^{-8}, 2.0 × 10^{-8}, and 3.7 × 10^{-8}, respectively), and post-hoc comparisons against the naïve group remained significant for Arg^+/Phe^- (P = 3.8 × 10^{-6}, 7.8 × 10^{-6}, 2.4 × 10^{-5}, 4.8 × 10^{-5}, and 8.0 × 10^{-5}), Phe^+/Arg^- (P = 8.4 × 10^{-7}, 1.1 × 10^{-6}, 1.1 × 10^{-6}, 9.1 × 10^{-7}, and 1.5 × 10^{-6}), but not Phe^+/Trp^- (P = 0.428, 0.284, 0.337, 0.261, and 0.275). P-values were adjusted for multiple comparisons by Bonferroni correction.

Extended Data Fig. 6c: After shuffling of manifold labels (juvenile fish), dE between manifolds representing two amino acids (AA↔AA), one amino acid and one bile acid (AA↔BA) and two bile acids (BA↔BA) were not significantly different in naïve (AA↔AA: 1.7 ± 0.2, mean ± SD, AA↔BA: 1.6 ± 0.2, BA↔BA: 1.3 ± 0.2) and trained (AA↔AA: 1.8 ± 0.4, Mann-Whitney U test, P = 0.20, AA↔BA: 1.6 ± 0.4, P = 0.52, BA↔BA: 1.3 ± 0.3, P = 0.45). All statistical tests were two-sided. P-values were not adjusted for multiple comparisons.

Extended Data Fig. 6f: After shuffling of manifold labels (juvenile fish), dM from an amino acid manifold to another amino acid manifold (AA→AA), from one bile acid manifold to another bile acid manifold (BA→BA), from one amino acid manifold to a bile acid manifold (AA→BA) and from a bile acid manifold to an amino acid manifold (BA→AA) in naïve (AA→AA: 39.2 ± 1.5, mean ± SD, AA→BA: 38.5 ± 1.5, BA→AA: 38.6 ± 1.5, BA→BA: 36.7 ± 1.5) and trained (AA→AA: 39.6 ± 2.3, Mann-Whitney U test, P = 0.19, AA→BA: 39.0 ± 2.3, P = 0.06, BA→AA: 39.0 ± 2.3, P = 0.19, BA→BA: 36.8 ± 2.0, P = 0.62) were not significantly different. All statistical tests were two-sided. P-values were not adjusted for multiple comparisons.

Extended Data Fig. 6i: After shuffling (adult fish), dE between CS+ and CS- (CS↔CS), between conditioned and non-conditioned (CS↔nonCS), and between non-conditioned odors (nonCS↔nonCS) were not significantly different in naïve (CS↔CS: 0.76 ± 0.25, mean ± SD, CS↔nonCS: 0.76 ± 0.20, nonCS↔nonCS: 0.77 ± 0.20) and trained (CS↔CS: 0.92 ± 0.18, Mann-Whitney U test, P = 0.24, CS↔nonCS: 0.87 ± 0.19, P = 0.07, nonCS↔nonCS: 0.83 ± 0.18, P = 0.67). Pale boxes show dE before shuffling, same as in Fig. 8h. All statistical tests were two-sided. P-values were not adjusted for multiple comparisons.

Extended Data Fig. 6l: After shuffling (adult fish), dM between CS+ and CS- (CS↔CS), from conditioned to non-conditioned (CS↔nonCS), from non-conditioned to conditioned (nonCS↔CS), and between non-conditioned odors (nonCS↔nonCS) were significantly higher in trained (CS↔CS: 53.3 ± 3.8, mean ± SD, CS↔nonCS: 54.1 ± 5.4, nonCS↔CS: 54.2 ± 6.0, nonCS↔nonCS: 54.9 ± 6.0) than in naïve (CS↔CS: 46.1 ± 9.5, Mann-Whitney U test, P = 0.018, CS↔nonCS: 47.1 ± 9.3, P = 0.003, nonCS↔CS: 46.9 ± 9.0, P = 0.003, nonCS↔nonCS: 48.3 ± 9.3, P = 0.022) fish. All statistical tests were two-sided. P-values were not adjusted for multiple comparisons.

Extended Data Fig. 7a: Effective radius: amino acids vs amino acids (AA-AA) [naïve] 1.01 ± 0.02 (mean ± SD), n = 18 pairs from N = 6 fish, [trained] mean ± SD 0.98 ± 0.03, n = 75 odor pairs from N = 25 fish, Mann–Whitney U test, P = 9.4 × 10^{-5}. Amino acids vs bile acids (AA-BA) [naïve] 0.97 ± 0.03, n = 54 pairs from N = 6 fish, [trained] 0.93 ± 0.03, n = 225 pairs from N = 25 fish, Mann–Whitney U test, P = 7.8 × 10^{-8}. Bile acids vs bile acids (BA-BA) [naïve] 1.02 ± 0.03, n = 18 pairs from N = 6 fish, [trained] 0.99 ± 0.03, n = 75 pairs from N = 25 fish, Mann–Whitney U test, P = 0.0005. Effective dimension: AA-AA [naïve] 18.1 ± 1.1, [trained] 17.1 ± 1.5, P = 0.002. AA-BA [naïve] 17.0 ± 1.5, [trained] 16.3 ± 1.6, P = 0.006. BA-BA

[naïve] 19.3 ± 0.9, [trained] 19.1 ± 1.5, P = 0.563. Effective center alignment: AA-AA [naïve] 0.86 ± 0.03, [trained] 0.84 ± 0.03, P = 0.015. AA-BA [naïve] 0.81 ± 0.04, [trained] 0.78 ± 0.04, P = 0.0002. BA-BA [naïve] 0.87 ± 0.03, [trained] 0.85 ± 0.03, P = 0.006. Effective axis alignment: AA-AA [naïve] mean ± SD 0.128 ± 0.033, [trained] 0.120 ± 0.022, P = 0.550. AA-BA [naïve] 0.098 ± 0.020, [trained] 0.089 ± 0.024, Mann–Whitney U test, P = 0.005. BA-BA [naïve] 0.121 ± 0.032, [trained] 0.106 ± 0.031, Mann–Whitney U test, P = 0.061. All statistical tests were two-sided. P-values were not adjusted for multiple comparisons.

Extended Data Fig. 7b: Percentage of capacity increase compared to the naïve group. Arg^+/Phe^-: Phe vs Arg: 11.55 ± 14.91, n = 9, P = 0.145. Phe vs Trp: 12.72 ± 13.96, n = 9, P = 0.050. Arg vs Trp: 11.40 ± 15.81, n = 9, P = 0.145. Phe^+/Arg^-: Phe vs Arg: 6.66 ± 7.32, n = 10, P = 0.118. Phe vs Trp: 9.87 ± 6.13, n = 10, P = 0.007. Arg vs Trp: 10.19 ± 5.49, n = 10, P = 0.005. Phe^+/Trp^-: Phe vs Arg: 11.30 ± 5.23, n = 6, P = 0.015. Phe vs Trp: 4.44 ± 5.59, n = 6, P = 0.180. Arg vs Trp: 8.99 ± 7.61, n = 6, P = 0.065. All statistical comparisons were performed using a Mann-Whitney U test (two-sided). P-values were not adjusted for multiple comparisons.

Extended Data Fig. 7c: For a sample size of 200 neurons, group differences were significant (Kruskal–Wallis test, n = 465 odor pairs from N = 31 fish, P = 6.5×10^{-6}), with post-hoc Dunn tests indicating higher manifold capacity relative to the naïve group for Arg^+/Phe^- (P = 9.2×10^{-7}), Phe^+/Arg^- (P = 0.010), and Phe^+/Trp^- (P = 0.049). For sample sizes of 300, 400, 500, 600, and 700 neurons, group differences remained significant (Kruskal–Wallis tests: P = 7.8×10^{-6}, 4.6×10^{-6}, 5.4×10^{-6}, 5.5×10^{-6}, and 3.0×10^{-6}, respectively). Post-hoc comparisons against the naïve group were consistently significant for Arg^+/Phe^- (P = 1.7×10^{-6}, 8.5×10^{-7}, 9.1×10^{-7}, 9.5×10^{-7}, and 5.4×10^{-7}) and Phe^+/Arg^- (P = 0.012, 0.011, 0.010, 0.013, and 0.009), whereas differences for Phe^+/Trp^- were marginal or weakly significant (P = 0.054, 0.059, 0.051, 0.057, and 0.049). P-values were adjusted for multiple comparisons by Bonferroni correction.

Extended Data Fig. 8: "Naïve" (random; mean ± SD): 0.066 ± 0.005, n = 120 odor pairs from N = 8 network configurations; "Trained" (with assemblies): 0.071 ± 0.009, n = 120 odor pairs from N = 8 network configurations (Mann–Whitney U test, P = 1.5×10^{-10}). Shuffled "naïve": 0.040 ± 0.0001; shuffled "trained": 0.040 ± 0.0001 (Mann–Whitney U test, P = 0.22). Effective radius was significantly lower for "trained" (mean ± SD 1.11 ± 0.05, n = 120 pairs from N = 8 network configurations) than for "naïve" networks (1.11 ± 0.05, n = 120 pairs from N = 8 network configurations; Mann–Whitney U test, P = 0.0002). Effective dimension was significantly lower for "trained" (22.9 ± 2.5) than for "naïve" networks (24.4 ± 1.5; Mann–Whitney U test, P = 2.2×10^{-11}). Effective center alignment was not significantly different ("trained": 0.45 ± 0.11; "naïve": 0.46 ± 0.10 Mann–Whitney U test, P = 0.084). Effective axis alignment was not significantly different ("trained": 0.048 ± 0.016; "naïve": 0.046 ± 0.011; Mann–Whitney U test, P = 0.60). All statistical tests were two-sided. P-values were not adjusted for multiple comparisons.

Extended Data Fig. 9b: Conditioned odors [mean ± SD], trained: 0.23 ± 0.55, n_{odor} = 2, n = 6092 neurons from N = 9 fish; naïve: 0.18 ± 0.49, n_{odor} = 2, n = 5164 neurons from N = 8 fish; Mann–Whitney U test, P = 7.6×10^{-88}; non-conditioned odors, trained: 0.22 ± 0.52, n_{odor} = 2, n = 6092 neurons from N = 9 fish; naïve: 0.19 ± 0.49, n_{odor} = 2, n = 5164 neurons from N = 8 fish; Mann–Whitney U test, P = 4.1×10^{-66}. All statistical tests were two-sided. P-values were not adjusted for multiple comparisons.

Extended Data Fig. 9f: dE between CS+ and CS- (CS↔CS) was significantly higher in trained (mean ± SD: 3.72 ± 0.98, n = 9 pairs from N = 9 fish) than in naïve fish (2.46 ± 0.98, n = 8 pairs from N = 8 fish, Mann–

Whitney U test, P = 0.046). dE between conditioned and non-conditioned (CS↔nonCS) was also significantly higher in trained (3.04 ± 1.03, n = 36 pairs from N = 9 fish) than in naïve fish (2.32 ± 0.86, n = 32 pairs from N = 8 fish, Mann–Whitney U test, P = 0.014). dE between non-conditioned odors (nonCS↔nonCS) was not significantly different (trained: 2.32 ± 0.66, n = 9 pairs from N = 9 fish; naïve: 2.01 ± 0.70, n = 8 pairs from N = 8 fish, Mann–Whitney U test, P = 0.423)**.** All statistical tests were two-sided. P-values were not adjusted for multiple comparisons.

Extended Data Fig. 9h: dM between conditioned odors (CS→CS) was higher in trained (247 ± 99, n = 18 pairs, from N = 9 fish) than in naïve (170 ± 52, n = 16 pairs, from N = 8 fish, Mann–Whitney U test, P = 0.012). dM from conditioned to non-conditioned odors (CS→nonCS) was higher in trained (237 ± 85, n = 36 pairs from N = 9 fish) than in naïve (166 ± 55, n = 32 pairs from N = 8 fish, Mann–Whitney U test, P = 2×10^{-4}). dM from non-conditioned to conditioned odors (nonCS→CS) was not significantly different (trained: 202 ± 64, n = 36 pairs from N = 9 fish; naïve: 173 ± 63, n = 32 pairs from N = 8 fish, Mann–Whitney U test, P = 0.072). dM from non-conditioned to non-conditioned odors (nonCS→nonCS) was not significantly different (trained: 194 ± 58, n = 18 pairs from N = 9 fish; naïve: 158 ± 52, n = 16 pairs from N = 8 fish, Mann–Whitney U test, P = 0.060). All statistical tests were two-sided. P-values were not adjusted for multiple comparisons.

Extended Data Fig. 9i: Capacity in trained fish (mean ± SD 0.081 ± 0.020, n = 54 pairs from N = 9 fish) was higher than in naïve fish (0.070 ± 0.017, n = 48 pairs from N = 8 fish; Mann–Whitney U test, P = 0.004). Differences after shuffling of labels were not significant (trained: 0.025 ± 0.0002; naïve: 0.025 ± 0.0002; Mann–Whitney U test, P = 0.8). All statistical tests were two-sided.

Extended Data Fig. 9j: Effective radius was significantly lower in trained (1.25 ± 0.11, n = 54 pairs from N = 9 fish) than in naïve fish (1.33 ± 0.10, n = 48 pairs from N = 8 fish; Mann–Whitney U test, P = 0.0006). Effective dimension was significantly lower in trained (17.8 ± 3.0) than in naïve fish (19.4 ± 3.7; Mann–Whitney U test, P = 0.032). Effective center alignment was lower in trained (0.89 ± 0.077) than in naïve fish (0.92 ± 0.051); Mann–Whitney U test, P = 0.013). Effective axis alignment was significantly lower in trained (0.207 ± 0.062) than in naïve fish (0.252 ± 0.084; Mann–Whitney U test, P = 0.004). All statistical tests were two-sided. P-values were not adjusted for multiple comparisons.

Extended Data Fig. 10a: Top left: Activity was significantly higher in all trained groups (Arg^+/Phe^-: 0.88 ± 0.94 [mean ± SD], n_{odor} = 3, n = 13013 neurons from N = 9 fish; Phe^+/Arg^-: 0.95 ± 0.93, n_{odor} = 3, n = 13379 neurons from N = 10 fish; Phe^+/Trp^-: 0.90 ± 0.93, n_{odor} = 3, n = 7760 neurons from N = 6 fish) than in naïve fish (0.81 ± 0.80, n_{odor} = 3, n = 9059 neurons from N = 6 fish; Kruskal–Wallis test, n = 129633, P = 4.2×10^{-111}). Posthoc Dunn's test: Arg^+/Phe^- vs naïve, P = 2.9×10^{-25}; Phe^+/Arg^- vs naïve, P = 3.5×10^{-102}; Phe^+/Trp^- vs naïve, P = 8.6×10^{-30}. P-values were adjusted for multiple comparisons by Bonferroni correction. Bottom, center: Naïve (mean ± SD): 0.134 ± 0.019, n = 90 odor pairs from N = 6 fish; trained: 0.145 ± 0.023, n = 375 odor pairs from N = 25 fish (Mann–Whitney U test, P = 1.2×10^{-5}). Shuffled naïve: 0.0200 ± 0.0001; shuffled trained: 0.0200 ± 0.0001 (Mann–Whitney U test, P = 0.89). All statistical tests were two-sided.

Extended Data Fig. 10b: Top left: Activity was significantly higher in all trained groups (Arg^+/Phe^-: 0.95 ± 1.06 [mean ± SD], n_{odor} = 3, n = 13013 neurons from N = 9 fish; Phe^+/Arg^-: 1.07 ± 1.10, n_{odor} = 3, n = 13379 neurons from N = 10 fish; Phe^+/Trp^-: 1.03 ± 1.11, n_{odor} = 3, n = 7760 neurons from N = 6 fish) than in naïve fish (0.90 ± 0.93, n_{odor} = 3, n = 9059 neurons from N = 6 fish; Kruskal–Wallis test, n = 129633, P = 1.7×10^{-140}). Posthoc Dunn's test: Arg^+/Phe^- vs naïve, P = 5.8×10^{-11}; Phe+/Arg- vs naïve, P = 1.4×10^{-104};

Phe^{+}/Trp^{-} vs naïve, $P = 4.0 \times 10^{-46}$. P-values were adjusted for multiple comparisons by Bonferroni correction. Bottom, center: Naïve (mean ± SD): 0.065 ± 0.008, n = 90 odor pairs from N = 6 fish; trained: 0.073 ± 0.014, n = 375 odor pairs from N = 25 fish (Mann–Whitney U test, $P = 6.9 \times 10^{-8}$). Shuffled naïve: $0.014 \pm 4.6 \times 10^{-5}$; shuffled trained: $0.014 \pm 5.5 \times 10^{-5}$ (Mann–Whitney U test, P = 0.59). All statistical tests were two-sided.

Extended Data Fig. 10c: Top left: Activity was significantly higher in all trained groups (Arg^{+}/Phe^{-}: 0.13 ± 0.21 [mean ± SD], $n_{odor} = 3$, n = 13013 neurons from N = 9 fish; Phe^{+}/Arg^{-}: 0.14 ± 0.19, $n_{odor} = 3$, n = 13379 neurons from N = 10 fish; Phe^{+}/Trp^{-}: 0.14 ± 0.21, $n_{odor} = 3$, n = 7760 neurons from N = 6 fish) than in naïve fish (0.11 ± 0.15, $n_{odor} = 3$, n = 9059 neurons from N = 6 fish; Kruskal–Wallis test, n = 129633, $P = 7.1 \times 10^{-82}$). Posthoc Dunn's test: Arg^{+}/Phe^{-} vs naïve, $P = 3.4 \times 10^{-25}$; Phe^{+}/Arg^{-} vs naïve, $P = 2.8 \times 10^{-72}$; Phe^{+}/Trp^{-} vs naïve, $P = 1.2 \times 10^{-45}$. P-values were adjusted for multiple comparisons by Bonferroni correction. Bottom, center: Naïve (mean ± SD): 0.043 ± 0.008, n = 90 odor pairs from N = 6 fish; trained: 0.049 ± 0.013, n = 375 odor pairs from N = 25 fish (Mann–Whitney U test, $P = 6.7 \times 10^{-5}$). Shuffled naïve: $0.014 \pm 4.1 \times 10^{-5}$; shuffled trained: $0.014 \pm 4.0 \times 10^{-5}$ (Mann–Whitney U test, P = 0.94). All statistical tests were two-sided.

SUPPLEMENTARY NOTE 3 | Manifold capacity analysis in the OB

In fish used for the analysis of odor-evoked activity in pDp (N = 31 total; Naïve: N = 6; Arg^+/Phe^-: N = 9; Phe^+/Arg^-: N = 10; Phe^+/Trp^-: N = 6), activity evoked by the same odors was also measured in the OB. An initial analysis of manifold capacity in the OB revealed that activity patterns evoked by different odors were often not linearly separable. To achieve linear separability, which is a requirement for manifold capacity analysis, enforced it by adding noise dimensions[1]. Under these conditions we observed slightly higher manifold capacity in trained as compared to naïve fish only when the analysis was focused on specific time windows. These effects were weaker than effects observed in pDp and not robust to variation of the analysis time window. We therefore conclude that effects of training on manifold capacity were more pronounced in pDp than in the OB. These results are consistent with the assumption that experience-dependent changes in representational manifolds are driven by network plasticity primarily in pDp, rather than in the OB. Moreover, these results support the notion that pDp generally supports pattern classification by enhancing linear separability of odor representations. However, further analyses are required to analyze effects of training on odor representations in the OB in more detail.

1 Chou, C.-N. *et al.* Neural Manifold Capacity Captures Representation Geometry, Correlations, and Task-Efficiency Across Species and Behaviors. *bioRxiv*, 2024.2002.2026.582157 (2024).

Settings	exc→exc	inh→exc	OB→exc	exc→inh	OB→inh	inh→inh
A	95	410	128	58	68	180
B	94	400	128	58	66	150
C	80	450	128	68	68	220
D	95	520	95	51	42	190

Supplementary Table 1. Weight strength parameter settings for computational modeling (unit: pS)
