# Supplementary material for: Representational learning by optimization of neural manifolds in an olfactory memory network
Source: Nat Neurosci. 2026 Sep 10;29(11):2810–21. doi: 10.1038/s41593-026-02429-3 (PMC13724268; doi:10.1038/s41593-026-02429-3)
Supplement: Supplementary file 2 [file 41593_2026_2429_MOESM2_ESM.pdf]

nature portfolio

Corresponding author(s): Rainer Friedrich

Last updated by author(s): Jul 9, 2026

Reporting Summary

Nature Portfolio wishes to improve the reproducibility of the work that we publish. This form provides structure for consistency and transparency in reporting. For further information on Nature Portfolio policies, see our Editorial Policies and the Editorial Policy Checklist.

Statistics

For all statistical analyses, confirm that the following items are present in the figure legend, table legend, main text, or Methods section.

n/a	Confirmed	
☐	☒	The exact sample size (*n*) for each experimental group/condition, given as a discrete number and unit of measurement
☐	☒	A statement on whether measurements were taken from distinct samples or whether the same sample was measured repeatedly
☐	☒	The statistical test(s) used AND whether they are one- or two-sided<br>*Only common tests should be described solely by name; describe more complex techniques in the Methods section.*
☐	☒	A description of all covariates tested
☐	☒	A description of any assumptions or corrections, such as tests of normality and adjustment for multiple comparisons
☐	☒	A full description of the statistical parameters including central tendency (e.g. means) or other basic estimates (e.g. regression coefficient) AND variation (e.g. standard deviation) or associated estimates of uncertainty (e.g. confidence intervals)
☐	☒	For null hypothesis testing, the test statistic (e.g. *F*, *t*, *r*) with confidence intervals, effect sizes, degrees of freedom and *P* value noted<br>*Give P values as exact values whenever suitable.*
☒	☐	For Bayesian analysis, information on the choice of priors and Markov chain Monte Carlo settings
☒	☐	For hierarchical and complex designs, identification of the appropriate level for tests and full reporting of outcomes
☐	☒	Estimates of effect sizes (e.g. Cohen's *d*, Pearson's *r*), indicating how they were calculated

Our web collection on statistics for biologists contains articles on many of the points above.

Software and code

Policy information about availability of computer code

Data collection	Image data were collected using scanimage (https://ch.mathworks.com/de/products/connections/product_detail/scanimage.html). Acquisition and first-order analysis of video data for behavioral experiments was performed using custom software as described (Namekawa I, Moenig NR, Friedrich RW, Rapid olfactory discrimination learning in adult zebrafish. Exp Brain Res 236:2959–2969, 2018).
Data analysis	Data were analyzed using code written in Matlab and Python. As stated in the data availability statement, code for simulations is available at https://github.com/clairemb90/pDp-model_juvenile and code for analysis is available at https://github.com/aloejhb/pDp_manifold_analysis and https://github.com/aloejhb/catrace. The code for manifold capacity analysis is provided as Supplementary Software (zip file).

For manuscripts utilizing custom algorithms or software that are central to the research but not yet described in published literature, software must be made available to editors and reviewers. We strongly encourage code deposition in a community repository (e.g. GitHub). See the Nature Portfolio guidelines for submitting code & software for further information.

Data

Policy information about availability of data

All manuscripts must include a data availability statement. This statement should provide the following information, where applicable:

- Accession codes, unique identifiers, or web links for publicly available datasets
- A description of any restrictions on data availability
- For clinical datasets or third party data, please ensure that the statement adheres to our policy

The experimental data and the simulation results that support the findings of this study are available in Figshare. doi: 10.6084/m9.figshare.28677233.

Research involving human participants, their data, or biological material

Policy information about studies with human participants or human data. See also policy information about sex, gender (identity/presentation), and sexual orientation and race, ethnicity and racism.

Reporting on sex and gender	N/A
Reporting on race, ethnicity, or other socially relevant groupings	N/A
Population characteristics	N/A
Recruitment	N/A
Ethics oversight	N/A

Note that full information on the approval of the study protocol must also be provided in the manuscript.

Field-specific reporting

Please select the one below that is the best fit for your research. If you are not sure, read the appropriate sections before making your selection.

☒ Life sciences ☐ Behavioural & social sciences ☐ Ecological, evolutionary & environmental sciences

For a reference copy of the document with all sections, see nature.com/documents/nr-reporting-summary-flat.pdf

Life sciences study design

All studies must disclose on these points even when the disclosure is negative.

Sample size	Sample sizes were not predetermined for this study. The number of animals per group was similar to other studies using a similar design. Subsampling was performed to verify that sample size was sufficient. The number of neurons per fish was maximized, approaching the total number of neurons (no sampling). A total of 31 independent imaging experiments (different fish) were performed for this study. The study comprised four experimental groups. Within each group, experiments were replicated at least six times (6, 9, 10, 6 fish per group).
Data exclusions	No data were excluded from analysis except for datasets compromised by obvious technical problems.
Replication	Reproducibility was confirmed by subsampling. In all cases, consistent results were obtained when analyses were performed repeatedly on random sub-samples.
Randomization	Animals were allocated to the same (different) experimental group when they received the same (different) behavioral training. Within each experimental group, analyses were randomized across animals and neurons.
Blinding	Experimenters were not blinded but all experiments and analyses were performed using standardized and automated procedures to minimize any potential bias.

Reporting for specific materials, systems and methods

We require information from authors about some types of materials, experimental systems and methods used in many studies. Here, indicate whether each material, system or method listed is relevant to your study. If you are not sure if a list item applies to your research, read the appropriate section before selecting a response.

Materials & experimental systems

n/a	Involved in the study
☒	☐ Antibodies
☒	☐ Eukaryotic cell lines
☒	☐ Palaeontology and archaeology
☐	☒ Animals and other organisms
☒	☐ Clinical data
☒	☐ Dual use research of concern
☒	☐ Plants

Methods

n/a	Involved in the study
☒	☐ ChIP-seq
☒	☐ Flow cytometry
☒	☐ MRI-based neuroimaging

Animals and other research organisms

Policy information about studies involving animals; ARRIVE guidelines recommended for reporting animal research, and Sex and Gender in Research

Laboratory animals	Zebrafish, juvenile and adult (28 days post fertilization and older). Genotypes: wild-type, Tg[alphaTubulin:GCaMP6s], or Tg[dlx4/6:ChR2-YFP]. Animals were raised in the facility of the Friedrich Miescher Institute for Biomedical Research. For details and references see Methods.
Wild animals	The study did not involve wild animals.
Reporting on sex	Animals were randomly chosen from both sexes to avoid any sex bias.
Field-collected samples	The study did not involve samples collected from the field
Ethics oversight	All experimental protocols were approved by the Veterinary Department of the Kanton Basel-Stadt (Switzerland).

Note that full information on the approval of the study protocol must also be provided in the manuscript.

Plants

Seed stocks	N/A
Novel plant genotypes	N/A
Authentication	N/A
