# Supplementary material for: Representational learning by optimization of neural manifolds in an olfactory memory network
Source: Nat Neurosci. 2026 Sep 10;29(11):2810–21. doi: 10.1038/s41593-026-02429-3 (PMC13724268; doi:10.1038/s41593-026-02429-3)

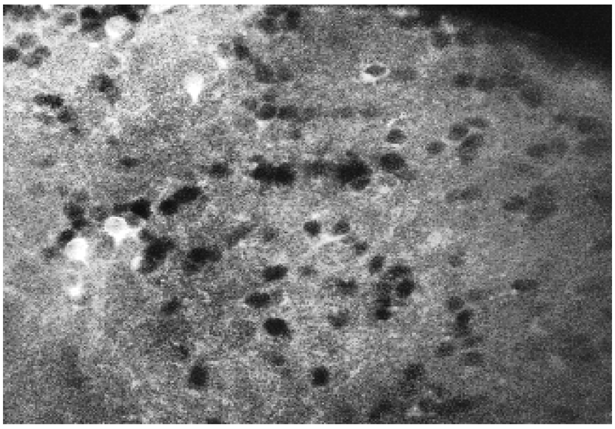

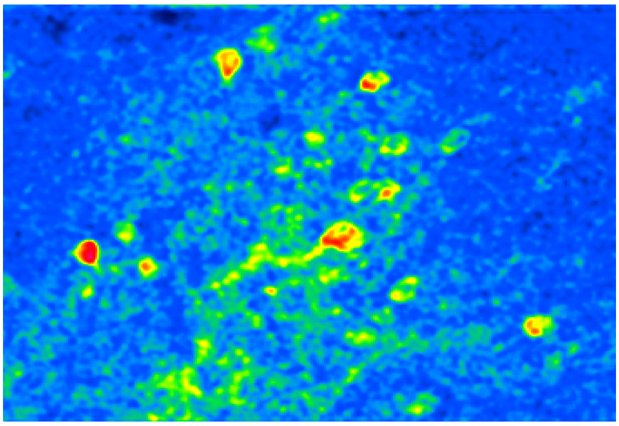

Supplement: Supplementary file 13 [file 41593_2026_2429_MOESM4_ESM.zip › figure01/Fig1b_images.pdf]
